# Prevalence of HPV Genotypes among Greek Women in Association with Their Potential to Cause Precancerous Lesions

**DOI:** 10.3390/microorganisms12071404

**Published:** 2024-07-11

**Authors:** Athanasia Kafasi, Georgios Kaparos, Vassiliki C. Pitiriga, Nikolaos Spanakis, Nikolaos Vlachos, Nikolaos Thomakos, Stamatios Stournaras, Athanasios Tsakris

**Affiliations:** 1 Deparment of Microbiology, Medical School, National and Kapodistrian University of Athens, 11527 Athens, Greece; 2 Department of Microbiology, Areteion Hospital, National and Kapodistrian University of Athens, 11528 Athens, Greece; 3 2nd Department of Obstetrics and Gynecology, Areteion Hospital, National and Kapodistrian University of Athens, 11528 Athens, Greece; 4 Division of Gynecologic Oncology, 1st Department of Obstetrics and Gynecology, Alexandra Hospital, National and Kapodistrian University of Athens, 11528 Athens, Greece; 5 Maternity and Gynecology Clinic, IASO Hospital, 15123 Athens, Greece

**Keywords:** HPV genotypes, human papillomavirus, cervical cancer, HPV vaccination, HPV51

## Abstract

The escalating global rates of precancerous lesions associated with human papillomavirus (HPV) types not targeted by current vaccines underscore the need to explore the prevalence of HPV types within the Greek female population and their involvement in precancerous lesion development. In the current study, we enrolled a cohort of 253 women aged 18 to 65 years, residing in Greece, who underwent routine screening in three tertiary care referral hospitals. Each participant completed a demographic questionnaire. An HPV DNA test was administered using the VisionArray^®^ HPV kit (ZytoVision GmbH) to qualitatively detect and genotype 41 clinically relevant HPV genotypes. Of all 253 women examined, 114 (45.1%) tested positive for HPV DNA. The primary type detected was HPV51 (high-risk), present in 21 women (8.3% of the total), followed by HPV54 (low-risk) in 17 women (6.7%); HPV16 (high-risk) ranked third, identified in 14 women (5.5%). Among the HPV-positive women, 65 were positive for high-risk HPV types (57% of HPV-positive women) and were referred for colposcopy and cervical biopsy. These procedures identified 24 women with cervical intraepithelial neoplasia 1 (CIN1) lesions and 2 with cervical intraepithelial neoplasia 2 (CIN2) lesions. The most prevalent HPV type among women with CIN1 lesions was HPV16, found in nine (37.5%) women, while HPV51 ranked second, identified in six (25%) women. Both women with CIN2 lesions tested positive for HPV16, whereas one of them was also tested positive for HPV45. Our study is the first to report the prevalence of HPV51 among HPV-positive women in the Greek female population. This highlights the need for further research to fully understand the potential of HPV types not covered by current vaccines, such as HPV51, to cause high-grade lesions or cervical cancer.

## 1. Introduction

In recent years, extensive efforts were made to decline the incidence of and mortality from cervical cancer through the vaccination of the young population against human papillomavirus (HPV) (primary prevention) and the simultaneous implementation of cervical cancer screening using the Papanicolaou (Pap) test or Liquid Based Cytology (LBC) in conjunction with sensitive molecular HPV DNA testing techniques (secondary prevention) [1]. However, cervical cancer remains a significant global health concern, ranking as the fourth most common cancer among women worldwide [2,3]. In 2022, around 660,000 women were diagnosed with cervical cancer, and around 350,000 women lost their lives to the disease [2,4,5,6]. In the same year, about 94% of the 350,000 deaths caused by cervical cancer occurred in low- and middle-income countries. Sub-Saharan Africa (SSA), Central America, and South-East Asia reported to have the highest rates of cervical cancer incidence and mortality [2,4,5,6].

Factors that are influencing the global spread of the disease are inequalities in access to vaccination, screening and treatment services, sexual behaviors including age of onset of sexual activity and number of sexual partners, access to precaution methods, human immunodeficiency virus (HIV) prevalence, smoking, and social and economic determinants such as gender biases and poverty [2,7,8]. Women living with HIV are six times more likely to develop cervical cancer compared to the general population, and 5% of all cervical cancer cases are estimated to be attributed to HIV [9].

Over 200 HPV types were described [10,11,12] and more than 40 types can be transmitted through sexual contact [12,13]. The prevalence of HPV infection is estimated between 9% and 13% of the world population [14,15]. Many HPV infections cause no symptoms and 90% resolve spontaneously within two years [10,12]. The oncogenic or high-risk HPV types (HR HPV) are: 16, 18, 31, 33, 35, 39, 45, 51, 52, 56, 58, and 59. About 70% of cervical cancer cases worldwide are attributed to HPV16 and HPV18 [16,17,18]. An individual can be infected with more than one type of HPV. In a study conducted in 2011 in Costa Rica, of the 2478 HPV-positive women, 1070 (43.2%) were infected with multiple types [19].

According to the Catalan Institute of Oncology and the International Agency for Research on Cancer (ICO/IARC) Information Centre on HPV and Cancer (2023), Greece has a population of 4.60 million women aged 15 years and older who are at risk of developing cervical cancer [20]. Current estimates suggest that every year, 697 women are diagnosed with cervical cancer, and 282 die from the disease [20]. Cervical cancer ranks as the 10th most frequent cancer among women in Greece and the 3rd most frequent cancer among women aged 15 to 44 years [20]. Approximately 2.8% of women in the general population are estimated to have a cervical HPV16/18 infection at any given time, and 52.3% of invasive cervical cancers are attributed to HPVs 16 or 18 [20]. However, the remaining 47.7% of invasive cervical cancers are caused by other HPV types than HPVs 16 and 18 and are often associated with HPV types not covered by available HPV vaccines. According to a study of 2417 women in Greece between 2011 and 2016, HPV prevalence was 43.9%, and HR HPV accounted for 31.3%. At the same study, HPV16 was the most common high-risk type followed by HPV51 and HPV31 [21].

These facts, along with evidence that vaccination programs can alter the epidemiology of diseases by changing the prevalence of different serotypes, underscore the need to investigate the current prevalence of HPV types in the Greek female population and their role in the development of low-grade and high-grade precancerous lesions.

## 2. Methods

### 2.1. Study Design

A population of women (aged between 18 and 65 years), consecutively attending the Areteion Hospital, Alexandra Hospital and IASO Hospital, for routine gynecological testing during the period of May 2019–February 2021, and permanently residing in Greece, participated in the study. Exclusion criteria included the following: age < 18 years or >65 years, pregnancy, hysterectomy, history of invasive treatment for CIN1 in the last 7 years, vaccination with the bivalent or nine-valent HPV vaccine, previous HPV DNA testing, High-Grade Squamous Intraepithelial Lesion (HGSIL) result in the last Pap test, HIV infection, immunosuppression, history of chemotherapy in the last 5 years, and history of warts infection. Prior to examination, each woman completed a demographic questionnaire concerning their age, area of residence in Greece, smoking, age of onset of their sexual activity, children and age of maternity, Pap test frequency, and HPV vaccination. Data on educational level, economic status, the number of sexual partners, and use of precautions during sexual activity were not available for the study.

The study comprised two phases. In the initial phase, cervical samples were obtained from all participants. Gynecologists utilized the digene^®^ hybrid capture 2 (HC2) DNA Collection Device (QIAGEN), which includes a cervical brush and a specimen transport medium, or a cervical brush combined with the CellSolutions™ General Cytology Preservative, for sample collection. Subsequently, the samples were transferred to the Virology Unit of the Medical School of Athens for HPV DNA genotyping of 41 HPV genotypes. Women testing positive for HPV DNA of one or more HR HPV types proceeded to the second phase, which involved a follow-up visit to the gynecologist for colposcopy and cervical biopsy. All participants were provided with detailed explanations of all procedures, and they consented by signing an informed document, agreeing to the anonymous processing of their results for the purpose of this study. The study was approved by the Bioethics and Ethics Committee of Medical School, National and Kapodistrian University of Athens (NKUA) (No: 1617023003/31.7.2017).

### 2.2. HPV DNA Genotyping

For HPV DNA genotyping, we used, according to the manufacturer’s instructions, the VisionArray^®^ HPV kit (ZytoVision GmbH, Bremerhaven, Germany) for the qualitative detection and genotyping of 41 clinically relevant HPV genotypes by DNA/DNA hybridization of Polymerase Chain Reaction (PCR) amplicons on immobilized complementary molecules, which were arranged on a glass chip. The first step was DNA extraction. For this purpose, we used the Maxwell^®^ 16 Viral Total Nucleic Acid Purification Kit (Promega) according to the manufacturer’s instructions, with the Maxwell^®^ 16 Instrument, which provides automated purification of viral total nucleic acid from 1 to 16 samples, at the elution volume of 50 μL. One ml of the preserved sample was transferred into an Eppendorf tube and was centrifuged down at 2000 rpm for 12 min. The supernatant was discharged and the pellet was resuspended in final volume of 300 μL, proceeding to the purification protocol of the Maxwell^®^ 16 Viral Total Nucleic Acid Purification Kit.

Five microlitres of DNA solution were used for the PCR step added to 15 μL of the HPV PreCise Master Mix, which is an improvement of the General Primer Mediated 5/6 (GP5/GP6) system and is used to amplify and biotinylate specific sections of the L1 region, a highly conserved region of the HPV genome, in a total reaction volume of 25 μL. The next step was the hybridization of the PCR product mixed with the hybridization solution onto the VisionArray^®^ HPV Chip 1.0, which contains immobilized DNA fragments of 41 HPV genotypes classified as: (a) high-risk: 16, 18, 31, 33, 35, 39, 45, 51, 52, 56, 58, and 59, (b) probably high-risk: 26, 34, 53, 66, 67, 68a, 68b, 69, 70, 73, 82IS39, and 82MM4, and (c) low-risk: 6, 11, 40, 42, 43, 44, 54, 55, 57, 61, 62, 72, 81CP8304, 83MM7, 84MM8, 90, and 91 [22,23]. The hybridization between the amplified sequences and the complementary DNA captures was performed subsequently. After the hybridization, the unspecifically bound DNA was washed away by short stringent wash steps. The specific bound biotinylated sequences were secondarily labeled with a streptavidin-peroxidase conjugate (detection solution) and visualized by a tetramethylbenzidine (TMB) staining (blue spot solution). The automated evaluation of the results is performed by the respective VisionArray^®^ analyzer software 2.0.

### 2.3. Colposcopy and Cervical Biopsy

All women who tested positive for DNA in one or more high-risk HPVs were referred to colposcopy and biopsy. During colposcopy, the physician uses a colposcope, which magnifies and illuminates the view of the cervix, vagina, and vulvar surface, to visually distinguish normal tissue from lesions. An acetic acid solution (3–5%) is applied to the surface of the cervix using cotton swabs to enhance the visualization of abnormal areas. Areas that become white (acetowhiteness) are considered for biopsy. A Lugol’s iodine solution may be applied to the cervix if no visible lesions are present to help identify abnormal areas. Following colposcopy, a punch biopsy is performed. During this procedure, small pieces of tissue are taken from the cervix using a medical device called “biopsy forceps”. One or more punch biopsies may be performed on different areas of the cervix. The tissue samples are then examined by a pathologist using a microscope to determine whether there is a precancerous lesion or cancer.

### 2.4. Statistical Analysis

All the data obtained from each woman, including the demographic questionnaire, HPV DNA results, and biopsy results, were transferred to the statistical program “Statistical Package for the Social Sciences” (SPSS Statistics) for analysis and correlation. Statistical correlations were conducted using the Pearson Chi-square (χ^2^) test, which assesses whether there is a statistically significant difference between the expected and observed frequencies among the categories of the variables.

## 3. Results

### 3.1. Demographics

Our study population comprised 253 women permanently residing in Greece, aged between 18 and 65 years old, who met the aforementioned inclusion criteria. The majority of the study population (84.6%) resided in Attica, while the remaining percentage resided in various regions across Greece (Peloponnese, Central Greece, Aegean Islands, Ionian Islands, Thessaly, Epirus, and Crete). Concerning HPV vaccination, 25.3% received the quadrivalent (4V) vaccine against HPV. Among the 64 vaccinated women, 53.1% were vaccinated after the initiation of their sexual activity, and 46.9% were vaccinated before. Finally, only six women from the total population were previously diagnosed with another sexually transmitted disease (STD) in the past. Among them, four had herpes simplex virus 2 (HSV2) infection, and two had chlamydia infection. Epidemiological characteristics of the study population are presented in Table 1.

### 3.2. HPV DNA Test Results

**Figure 1:**
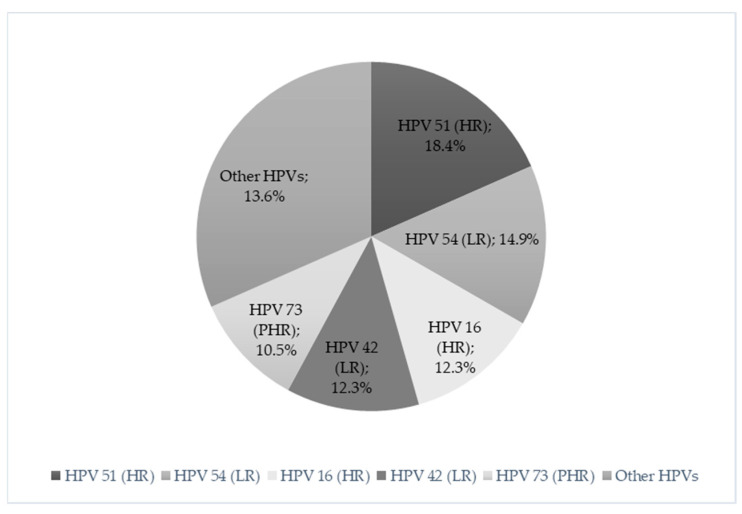
HPV types detected among HPV-positive women (*n* = 114). HR: High-Risk; PHR: Probably High-Risk; and LR: Low-Risk.

Out of 253 women, a total of 114 women (45.1%) tested positive for HPV DNA. The predominant type was HPV51 (high-risk), found in 21 women (8.3% of the total population), followed by HPV54 (low-risk), found in 17 women (6.7%). HPV16 (high-risk) and HPV42 (low-risk) came third, found in 14 women each (5.5%), followed by HPV 73 (probably high-risk), found in 12 women (4.7%). In Figure 1, the most frequent HPV types detected among all HPV-positive women are presented.

**Figure 2:**
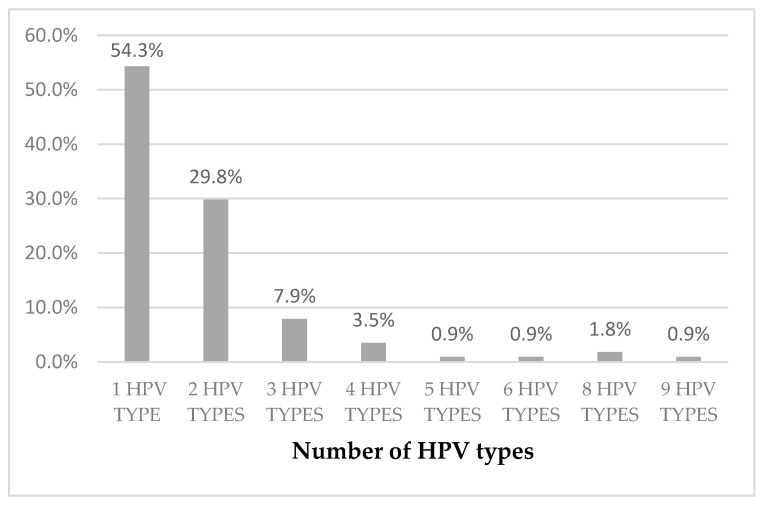
Percentages of HPV-positive women infected by one or more HPV types (*n* = 114).

Out of 114 HPV DNA positive women, 52 women (45.7%) were infected by more than one HPV type. In Figure 2, we present the percentages of women infected by one or more HPV types.

The most frequently encountered HR HPVs were: (1) HPV 51 (32.3% of HR HPV-positive women), (2) HPV16 (21.5%), (3) HPV18 and HPV59 (12.3%), and (4) HPV56 (10.7%). Among all HR HPV types found, the corresponding frequencies were: (1) HPV51 (25% of the total HR HPVs found), (2) HPV16 (16.7%), (3) HPV18 and HPV59 (9.5%), and (4) HPV56 (8.3%). These 65 women who tested positive for HR HPV underwent colposcopy and biopsy.

### 3.3. Biopsy Results

Out of the 65 women (25.7%) referred for colposcopy and biopsy, 39 women (15.4% of the total sample) had a normal result, 24 women (9.5% of the total sample) had a biopsy result indicating CIN1, and 2 women (0.8% of the total sample) were diagnosed with a CIN2 lesion. The five most frequent HPV types in the 24 women with CIN1 lesions were: HPV16 (high-risk), found in 9 women (37.5%), with 2 of them (8.3%) having a single infection and 7 of them (29.2%) having coinfections (4 with other HR HPVs and 3 with non-HR HPVs). HPV51 (high-risk) was found in six women (25%), all of whom had coinfections (three with other HR HPVs and three with non-HR HPVs). HPV73 (probably high-risk) was found in five women (20.8%), all with coinfections with HR HPVs, and HPV 31 (high-risk) was also found in five women (20.8%), with two of them (8.3%) having a single infection and three of them (12.5%) having coinfections with other HR HPVs. Finally, HPV56 (high-risk) was found in four women (16.7%), with one of them (4.2%) having a single infection and three of them (12.5%) having coinfections (two with other HR HPVs and one with non-HR HPVs). All HPV types detected in women with CIN1 lesions are presented in Table 2. In the two women with CIN2 lesions, the detected HPV types were as follows: firstly, HPV16 (high-risk) was found in both women (100%), with one of them (50%) having a single infection and the other (50%) having a coinfection with HPV45 (high-risk). Secondly, HPV45 (high-risk) was found in one woman (50%) as a coinfection with HPV16 (high-risk).

## 4. Discussion

Prior research [24] indicates that while the incidence of precancerous lesions associated with HPV strains targeted by the Cervarix vaccine (HPV16,18) significantly declined, there was a notable rise in precancerous lesions linked to HPV strains not covered by the vaccine. This increase is particularly evident in the vaccinated population compared to the unvaccinated, suggesting a limited effectiveness of the vaccine against HR HPV types not included in its formulation. Given this context, it is imperative to assess the prevalence of HPV strains among Greek women, alongside an investigation into their role in the development of both low-grade and high-grade precancerous lesions. This research becomes especially critical amidst ongoing changes in vaccination coverage due to the implementation of the ninth-valent (9V) vaccine in the Greek national vaccination program.

Upon analyzing the HPV DNA test results, we found a notably high prevalence of HPV, reaching 45%, with 25.7% testing positive for one or more HR HPV types. This percentage is elevated compared to findings from previous large studies that reported HR HPV rates ranging from 15.1% to 17.7% [25,26,27]. In a previous large study in China, the prevalence of HR HPV types was even lower at 8.8% [28]. Additionally, according to a meta-analysis, the overall infection rate of HR HPV in mainland Chinese women was 19.0% [29].

The high prevalence rates of HR HPVs can be attributed to several factors. Firstly, the low vaccination coverage, affecting only 25% of all women in our study, has a substantial impact. Another important factor is that a notable portion of our study sample received vaccination after they began engaging in sexual activity. The age distribution among our participants was a crucial aspect influencing our findings. A significant proportion of individuals in our study were between 18 and 30 years old, with another notable group aged 31 to 40 years. This differs from the ATHENA study [25], which included a smaller proportion of participants in the respective age groups. When correlating age with the DNA test results, we observed a statistically significant difference in the distribution of positive outcomes across age groups, particularly noting higher rates among younger individuals. This trend is consistent with findings from the ATHENA study [25].

Another contributing factor to the high HPV prevalence was the age at which individuals commence sexual activity, marking the onset of HPV infection. It is noteworthy that a substantial proportion of women in our study, amounting to 35%, initiated sexual activity between the ages of 12 and 17 years. A similar age stratification concerning the initiation of sexual activity was also documented in another Greek study [30].

Among the HPV types identified in our study, HPV51 was the most common, affecting 8.3% of the participants and comprising a quarter of all HR types detected. Not included in the available 9-valent (9V) vaccine due to its absence among the top ten HPV types detected in cervical cancers globally according to ICO/IARC [3,4,5,6], globally this virus ranks sixth among women with a normal Pap test, third in low-grade dysplasias, and seventh in high-grade dysplasias [3,4,5,6]. However, in Greece, HPV51 is notably the second most common HPV among women with low-grade dysplasia, fourth among those with high-grade lesions, and fifth in cervical cancers [20]. While the benefit of vaccination is evident in preventing infections from the HPVs included in the 9V vaccine, our study demonstrates that there is a “clinical unmasking” with HPV vaccination, meaning that protection against the strains covered by the vaccine leaves room for other high-risk HPVs, such as HPV51, to become more prevalent.

In our study, following HPV51 were HPV16, HPV18, HPV59, and HPV56. In the PIPAVIR study, HPV16 had the highest prevalence, followed by HPV51 [26]. Similarly, in the ARTISTIC study [31], the rates were lower, but HPV51 was the second most prevalent after HPV16. In a 2023 study in Iran, the most prevalent HR HPV types were HPV31, HPV16, HPV51, HPV18, and HPV66 [32]. In a 2022 study in South Africa, the dominant HPV types were HPV16, followed by HPV35, HPV58, HPV45, and HPV18 [33]. In a 2020 study in China, the most prevalent HR HPV genotypes were HPV52, HPV16, HPV58, HPV53, HPV39, and HPV51 [34].

In our study we observed a high prevalence of HR HPV and CIN1 lesions, with only a small number of women having CIN2 and CIN3/CIN3+ lesions. Research indicates that women with biopsy-proven CIN1 due to HR HPV have a low 5-year risk (~2%) of developing CIN3+ high-grade precancerous lesions [35]. However, the accuracy of lesion characterization relies heavily on the evaluator’s experience and skills. As a result, over 10% of CIN1 diagnoses eventually progress to CIN2+ lesions [36]. Therefore, we believe it is crucial to study the HPV types involved in causing CIN1 lesions.

When comparing our findings regarding the prevalence of CIN1 and CIN2 lesions with those of previous studies [25,27], we note similar percentages concerning CIN1 lesions. However, there is a disparity in the detection rates of CIN2 lesions, as our research indicates a lower occurrence compared to both the ATHENA study and the IMPACT trial [25,27].

It is noteworthy that none of the women included in our study exhibited CIN3 lesions, in contrast to findings from other studies [25,27]. This difference can be attributed to the high level of compliance among women in our sample with annual Pap testing. This screening regimen reduces the likelihood of overlooking cytological abnormalities indicative of CIN3 and possibly CIN2. Additionally, the exclusion criterion of women with a history of HGSIL cytology contributes to the lower incidence observed in our study.

Analyzing the results of our study concerning the HPV types profile and its relation with CIN lesions, we observe further differences to data from IARC [3,4,5,6]. Globally, HPV16 has a rate of 19.3% in CIN1 lesions and 45.1% in CIN2 lesions [3,4,5,6]. However, in Greece, HPV16 appears to not exceed 15% in CIN1 lesions and 44% in CIN2 lesions [20]. A meta-analysis of twenty-nine studies from Africa and seventeen studies from Asia identified the most prevalent HPV types in cervical malignancies. In Africa, the prevalent HPV types were HPV16, HPV52, HPV35, HPV18, and HPV58. In Asia, the prevalent types were HPV16, HPV52, HPV58, HPV33, and HPV53, in descending order of rank [37].

In a hypothetical scenario where all women were vaccinated with the 9V vaccine before initiating sexual activity, 6 out of 16 women who tested positive for HPVs 16 and 18, without any other high-risk types would have avoided a CIN1 lesion, and 1 a CIN2 lesion, sparing them from more frequent checks that evoke a high emotional burden.

In this hypothetical scenario, it is also noteworthy to examine the implications of the remaining five HR HPV types, 31, 33, 45, 52, and 58 covered by this newer vaccine, in CIN1 lesions.

Regarding HPV31, we observed that five cases of CIN1 were able to be prevented. However, concerning HPV33, its involvement in causing CIN1 lesions appears to be non-existent.

According to data from ICO/IARC, the prevalence of HPV33 is extremely low in women with a normal Pap test or CIN1. Nonetheless, this scenario changes in CIN2+ lesions and cancers, where HPV33 now ranks fifth and fourth in frequency in Greece and globally, respectively [3,4,5,6,20]. For this reason, its inclusion in the vaccination scheme was deemed necessary. The high-risk associated with HPV33 is further supported by a study conducted by Cuzick J., where he characterizes HPV33 as “very high-risk” due to its significantly high positive predictive factor. Consequently, it should be treated similarly to HPV16 [38].

Regarding HPV45 and HPV58, a total of four cases of CIN1 were able to be avoided. The reason why the 9V vaccine covers HPVs 45 and 58 is that although their prevalence in causing low-grade and high-grade lesions is low in Greece, globally, these two viruses are among the top five causes of cervical cancers [3,4,5,6,20].

Finally, the theoretical coverage of the female population in our sample against HPV52 seems to be low since no HPV52-positive cases exhibited lesions ≥ CIN1, a fact that also aligns with statistical data from Greece showing almost zero involvement of HPV52 in causing cervical lesions. This fact contrasts with what is occurring in developed countries, where HPV52 ranks seventh in cervical cancers, third in high-grade lesions, and fifth in low-grade dysplasias [3,4,5,6,20].

One notable limitation of our study is the relatively small sample size of women enrolled, resulting from the application of several exclusion criteria. These stringent criteria ultimately reduced the number of women eligible for inclusion. Another significant limitation is the absence of a control group consisting of women with normal Pap test results and negative HPV DNA test results. Such a control cohort would allow for a direct comparison of colposcopy and biopsy findings between the control group and the study participants.

## 5. Conclusions

Our research reveals a high prevalence of HPV51, which is not covered by the available vaccines. HPV51 emerged as the most prevalent HPV type among high-risk HPVs in our sample and was the second most common in biopsies presenting CIN1 lesions. The rates of HPV51 in Greek women presenting normal cytology, low-grade and high-grade lesions, and also cervical cancer are higher compared to the global averages. This underscores the need for continuous updating on HPV type prevalence, particularly focusing on HPV types not covered by the 9-valent vaccine, given the evolving vaccination coverage. Moreover, since the accuracy of lesion characterization heavily relies on the evaluator’s experience and skills, it is crucial to study the HPV types involved in causing CIN1 lesions to improve diagnosis and treatment outcomes.

## Figures and Tables

**Table 1:** Epidemiological characteristics of study population.

Characteristics of Study Population	Number of Women	Percentage (%)
Age		
18–30	95	37.5%
31–40	83	32.8%
41–50	55	21.7%
51–65	20	7.9%
Smoking		
Non-smokers	166	65.6%
Regular smokers	82	32.4%
Occasional smokers	5	2.0%
Age of first sexual contact		
12–17	88	34.8%
18–23	155	61.3%
24+	10	4.0%
Number of children		
0	150	59.3%
1	42	16.6%
2	48	19.0%
3	11	4.3%
4	2	0.8%
Age of first birth (for 103 women)		
<20	5	4.9%
20–29	46	44.7%
30–39	52	50.5%
Age of the first Pap test		
<20	167	66.0%
20–29	80	31.6%
30–39	5	2.0%
40+	1	0.4%
Frequency of Pap test		
Every 6 months	33	13.0%
Every 1 year	191	75.5%
Every 2–5 years	24	9.5%
Every 6+ years	5	2.0%
HPV vaccination (4V vaccine)		
Vaccinated	189	74.7%
Non-vaccinated	64	25.3%
Age of HPV vaccination		
12–16	14	21.9%
17–21	28	43.7%
22–26	17	26.6%
27+	5	7.8%
Alcohol history		
Yes	0	0.0%
No	253	100%
Menopausal state		
Pre-menopausal	224	88.5%
Post-menopausal	29	11.5%

**Table 2:** HPV types detected in women with CIN1 lesions (*n* = 24).

Hpv Types	Number of Women	Single Infection (%)	Multiple Infection (%)	Total Percentage
HPV16 (HR)	9	2 (8.3%)	7 (29.2%)	37.5%
HPV51 (HR)	6	0	6 (25%)	25%
HPV73 (PHR)	5	0	5 (20.8%)	20.8%
HPV31 (HR)	5	2 (8.3%)	3 (12.5%)	20.8%
HPV56 (HR)	4	1 (4.2%)	3 (12.5%)	16.7%
HPV90 (LR)	4	0	4 (16.7%)	16.7%
HPV67 (PHR)	3	0	3 (12.5%)	12.5%
HPV58 (HR)	3	1 (4.2%)	2 (8.3%)	12.5%
HPV66 (PHR)	3	0	3 (12.5%)	12.5%
HPV35 (HR)	2	0	2 (8.3%)	8.3%
HPV42 (LR)	2	0	2 (8.3%)	8.3%
HPV44 (LR)	2	0	2 (8.3%)	8.3%
HPV45 (HR)	2	0	2 (8.3%)	8.3%
HPV59 (HR)	2	0	2 (8.3%)	8.3%
HPV62 (LR)	2	0	2 (8.3%)	8.3%
HPV34 (PHR)	1	0	1 (4.2%)	4.2%
HPV6 (LR)	1	0	1 (4.2%)	4.2%
HPV18 (HR)	1	0	1 (4.2%)	4.2%
HPV52 (HR)	1	0	1 (4.2%)	4.2%
HPV53 (PHR)	1	0	1 (4.2%)	4.2%
HPV54 (LR)	1	0	1 (4.2%)	4.2%
HPV55 (LR)	1	0	1 (4.2%)	4.2%
HPV61 (LR)	1	0	1 (4.2%)	4.2%
HPV68 (PHR)	1	0	1 (4.2%)	4.2%
HPV70 (PHR)	1	0	1 (4.2%)	4.2%
HPV82 (LR)	1	0	1 (4.2%)	4.2%
HPV83 (LR)	1	0	1 (4.2%)	4.2%
HPV91 (LR)	1	0	1 (4.2%)	4.2%

HR: High-Risk; PHR: Probably High-Risk; and LR: Low-Risk.

## Data Availability

The original contributions presented in the study are included in the article, further inquiries can be directed to the corresponding author.
